# Vaccine Hesitancy and Anti-Vaccination Attitudes during the Start of COVID-19 Vaccination Program: A Content Analysis on Twitter Data

**DOI:** 10.3390/vaccines10020161

**Published:** 2022-01-21

**Authors:** Hüseyin Küçükali, Ömer Ataç, Ayşe Seval Palteki, Ayşe Zülal Tokaç, Osman Hayran

**Affiliations:** Department of Public Health, School of Medicine, Istanbul Medipol University, Istanbul 34815, Turkey; oatac@medipol.edu.tr (Ö.A.); aspalteki@medipol.edu.tr (A.S.P.); ayse.tokac@medipol.edu.tr (A.Z.T.); ohayran@medipol.edu.tr (O.H.)

**Keywords:** COVID-19, vaccines, vaccine hesitancy, Twitter, social media, content analysis

## Abstract

Twitter is a useful source for detecting anti-vaccine content due to the increasing prevalence of these arguments on social media. We aimed to identify the prominent themes about vaccine hesitancy and refusal on social media posts in Turkish during the COVID-19 pandemic. In this qualitative study, we collected public tweets (*n* = 551,245) that contained a vaccine-related keyword and had been published between 9 December 2020 and 8 January 2021 through the Twitter API. A random sample of tweets (*n* = 1041) was selected and analyzed by four researchers with the content analysis method. We found that 90.5% of the tweets were about vaccines, 22.6% (*n* = 213) of the tweets mentioned at least one COVID-19 vaccine by name, and the most frequently mentioned COVID-19 vaccine was CoronaVac (51.2%). We found that 22.0% (*n* = 207) of the tweets included at least one anti-vaccination theme. Poor scientific processes (21.7%), conspiracy theories (16.4%), and suspicions towards manufacturers (15.5%) were the most frequently mentioned themes. The most co-occurring themes were “poor scientific process” with “suspicion towards manufacturers” (*n* = 9), and “suspicion towards health authorities” (*n* = 5). This study may be helpful for health managers, assisting them to identify the major concerns of the population and organize preventive measures through the significant role of social media in early spread of information about vaccine hesitancy and anti-vaccination attitudes.

## 1. Introduction

The World Health Organization (WHO)’s Strategic Advisory Group of Experts on Immunization defines vaccine hesitancy as a “delay in acceptance or refusal of vaccines despite availability of vaccination services” [1]. Although vaccination is the key prevention strategy against communicable diseases, vaccine hesitancy is becoming more prevalent and is identified as one of the top ten threats to global health by the WHO [2]. Vaccine-hesitant individuals are not homogeneous and their attitudes, ranging from partial acceptance to complete refusal of various vaccines, are influenced by a variety of factors. Determinants of vaccine hesitancy have been classified into three categories, contextual influences (socio-economic, cultural, political, health system factors, etc.), individual and group influences (knowledge, beliefs, attitudes, experiences, etc.), and vaccine/vaccination-specific issues (costs, etc.). Each of these determinants can act as a barrier against vaccine hesitancy or promote such attitudes [1,3].

Current research on the acceptance and refusal of the COVID-19 vaccine shows that although attitudes vary by population, hesitancy is a universal problem. Studies identify fear of the vaccine’s side effects, skepticism about its safety, the short duration of immunity, doubts about its necessity and effectiveness, a lack of information, and general vaccine refusal as factors influencing acceptance or hesitancy. More broadly, an important finding is that the rate of acceptance of the COVID-19 vaccine rate has declined over time [4].

In recent decades, publicly posted materials on social media have become valuable sources for identifying the general public’s beliefs and attitudes towards important health issues, as well as understanding socio-cultural contexts [5]. Twitter is a leading social media platform, as it hosts real-time sharing of opinions, emotions, and content, and its large body of users provides access to broad populations. Data from these sources are used for content analysis, network analysis, surveillance, and even public health interventions [6].

As vaccination is a prominent issue in the public eye, online activity pertaining to vaccination-related attitudes and information-seeking behaviors about vaccination also take place via social media in a myriad of ways. According to a study which analyzed the contents of the most frequently visited vaccine-focused blogs and forums, non-expert-moderated sites contained more controversial information and more negative attitudes toward vaccination than expert-moderated sites [7].

In a study analyzing the sentiments of vaccine-related tweets between 2011 and 2019, it was found that both positive and negative sentiments in posts have increased in number and proportion over time compared to neutral sentiments. This increase is particularly noticeable as sentiment spikes in tweets about vaccinations during discussions about vaccines in traditional media [8]. Although the number of users creating anti-vaccine content on social media is smaller than the total number of users who share vaccine-related content, their online presence is becoming more prevalent and the amount of interactions with their content is increasing [9]. The content of anti-vaccine posts often includes personal stories, negative health impacts attributed to vaccination, discussions about vaccine ingredients, distrust of the pharmaceutical industry, criticism of vaccine research, political debates, and conspiracy theories [9,10].

Vaccine hesitancy is a growing problem in Turkey, despite high childhood vaccine coverage [11,12]. Lack of information on vaccines and distrust about the side effects, safety, and effectiveness of vaccines are all important factors in vaccine acceptance in Turkey, as they are throughout the world [13]. Despite the bans applied by social media platforms, anti-vaccine or vaccine-hesitant content posted in Turkish can be found on various social media platforms. A small number of studies have shown that vaccine-hesitant content involves arguments mainly relating to religious beliefs, in addition to distrust of the pharmaceutical industry and policymakers [14,15].

During the pandemic period, the increasing visibility and popularity of vaccine-hesitant content on social media is also a growing public health concern, threatening the public acceptance of newly developed COVID-19 vaccines [16]. Therefore, investigating anti-vaccine content on Twitter, a social media site where information and sentiment sharing about COVID-19 is high, may thus be beneficial for obtaining more detailed information about the subject during the pandemic. As anti-vaccine and vaccine-hesitant contents are not censored on social media in Turkey, this investigation may also help guide future interventions. In this study, we aimed to identify the prominent themes about vaccine hesitancy and refusal on Twitter during the start of COVID-19 vaccination program in Turkey.

## 2. Materials and Methods

This was a qualitative study with a content analysis design. We used Twitter as a social media platform of interest because it is one of the most widely used social media platforms in Turkey and it is more convenient for data collection than others. Although detailed user demographics are not available, according to a global social media report [17], 72.5% of internet users aged 16–64 have used Twitter in the past month and 77.7% of the Turkish population uses the internet.

Turkey imported the Sinovac (Sinovac Biotech Ltd., Beijing, China) vaccine (CoronaVac) from China and the first batch of vaccines arrived on 30 December 2020. We collected content related to vaccines published publicly on Twitter in Turkish between 9 December 2020 and 8 January 2021. Data were collected via software developed by a researcher in the Python programming language using open-source libraries and the Twitter Application Programming Interface (API) [18]. Instead of accessing all tweets in a particular day, Twitter allows users only to access tweets that contain user-provided keywords. Our software sends a request to Twitter using a search query and receives data of which the contents match the query. The query used in this study was composed of the keyword “vaccine” (“aşı” in Turkish) and its derivatives according to Turkish grammar. The complete search query can be found in File S1 Supplementary Material. Due to the restrictions of the standard Twitter API, data were collected on a weekly basis. In total, 551,245 tweets were collected in the study period.

In this qualitative study, our purpose was discovering the diversity of anti-vaccine themes, rather than generalizability. Although there is no accepted method in the literature for sample size determination specific to social media qualitative content analysis, data saturation is seen as a “gold standard” for qualitative studies and it is assessed during the data analysis [19]. We specified that an initial sample size of 1000 tweets could be adequate to reach data saturation and feasible for manual coding. If this was not sufficient to reach data saturation, we decided to sample further batches of 500 tweets until reaching saturation (the method used for the assessment of data saturation is explained below). Taking potential daily differences in a variety of tweet contents into account, the sample was selected randomly from each day in proportion with the number of tweets on that particular day. Due to rounding, the final study sample contained 1041 tweets.

Data for the following variables were collected for each tweet and their publishers: text, publication time, presence of visual content (picture or video), presence of a URL, presence of a hashtag; publisher’s duration of Twitter use, number of followers, number of tweets, and account verification status.

Four researchers independently analyzed and coded 260 tweets. Researchers applied an inductive coding method for anti-vaccine arguments in the contents. In addition, we extracted data from content for other (objective) variables using a simple coding framework that involved relevancy with the vaccine (homonymous or metaphorical use was considered irrelevant), type of the user (organizational or personal), and how the vaccine was named (if available).

**Figure 1:**
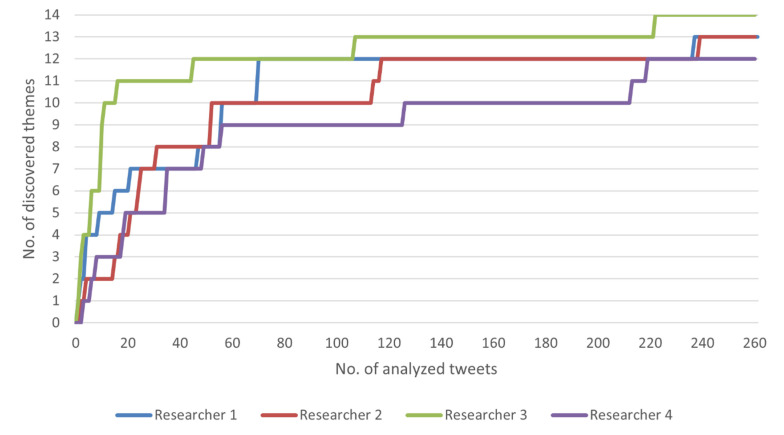
Data saturation assessment.

During the data analysis, data saturation was assessed retrospectively in two meetings using the methodology suggested by Guest et al. [20]. Figure 1 shows the number of new themes identified by the number of tweets analyzed for each researcher. As can be seen in the figure, there was only marginal information gain (2 out of 14 themes) after approximately 60 tweets has been analyzed and potentially discovering one more theme would cost approximately a thousand new tweets to be analyzed. In the second meeting, all researchers agreed that further sampling was not required and not efficient enough given the small likelihood of discovering new information.

After the completion of the independent analyses, inductive codes regarding anti-vaccine arguments were gathered in an online whiteboard, discussed, and synthesized, and emerging themes were identified based on a consensus among the researchers. Emerging themes are reported with example quotations. We translated the quoted tweets into English, while trying to conserve their informal nature and the syntax, which was not necessarily correct. Original Turkish texts of quotations can be found in File S2 Supplementary Material. The main qualitative findings are supported by descriptive statistics, presented as numbers and percentages for categorical variables and medians and interquartile ranges for continuous variables that are not distributed normally.

## 3. Results

### 3.1. Descriptive Statistics

The descriptive characteristics of the user accounts and the tweets are presented in Table 1 and Table 2, respectively. All tweets were published from 1000 unique accounts, of which 2.7% were verified and 11.3% were organizational users (Table 1). Among 1041 tweets included in the study, 90.5% were about vaccines, 11.1% included at least one visual, and 21.0% included a URL (Table 2).

**Figure 2:**
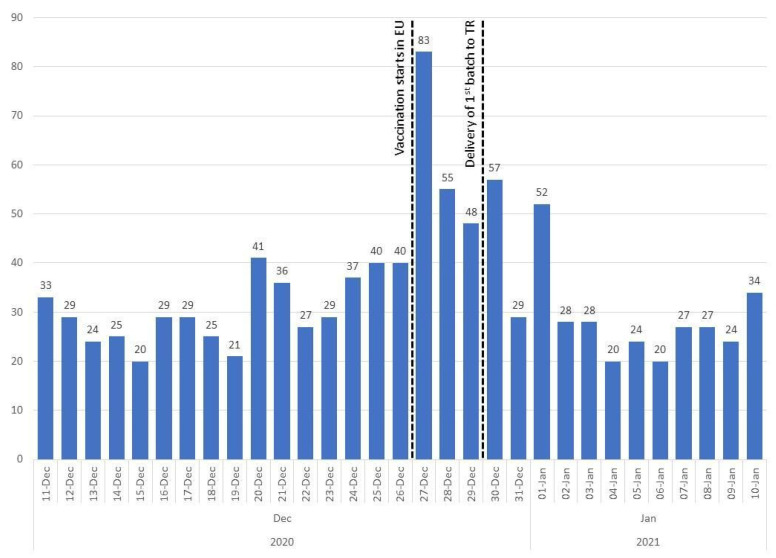
Daily numbers of tweets in the sample.

Daily numbers of tweets are shown in Figure 2. The median number of included tweets per day was 29.0 during 11–29 December which corresponds to the time before the arrival of the first group of vaccines to Turkey.

The content characteristics of the tweets are summarized in Table 3. As can be seen in Table 3, 22.6% (*n* = 213) of the tweets included at least one name of a COVID-19 vaccine; 22.0% (*n* = 207) of the tweets included at least one anti-vaccination theme.

The frequency distributions of COVID-19 vaccine names and anti-vaccination themes are presented in Table 4 and Table 5, respectively.

As can be seen in Table 4, 213 tweets included a total of 235 COVID-19 vaccine names. The most frequently mentioned COVID-19 vaccine was CoronaVac (51.2%). However, it was mostly expressed as “the Chinese vaccine” (42.3%).

### 3.2. Emerging Themes

Among all tweets, 22.0% (*n* = 207) had an anti-vaccination theme. In total, 207 tweets included 295 themes (56 tweets included two, 13 tweets include three, and two tweets included four themes.). The anti-vaccination themes that emerged in the contents of the tweets are detailed in Table 5.

Among tweets that included an anti-vaccination theme, suspicion of poor scientific processes (21.7%), conspiracy theories (16.4%), and suspicions towards manufacturers (15.5%) were the most frequently mentioned themes.

**Figure 3:**
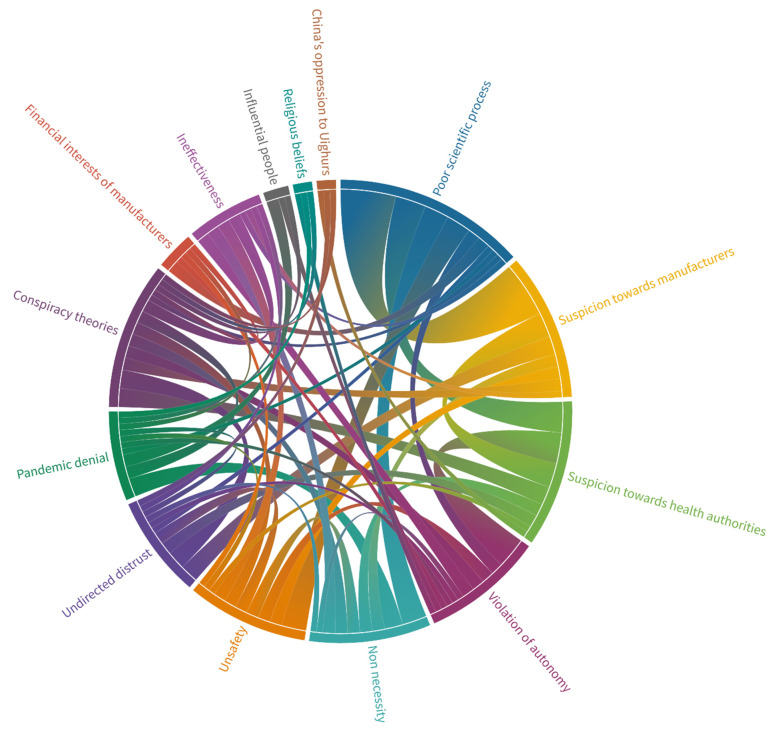
Co-occurrence of themes among tweets that contained more than one theme.

Seventy-one tweets (34.2%) were coded for more than one theme. The co-occurrence of themes among those tweets is visualized in Figure 3. Most commonly, the “poor scientific process” theme accompanied “suspicion towards manufacturers” (*n* = 9) and “suspicion towards health authorities” (*n* = 5).

#### 3.2.1. Poor Scientific Process

Suspicions about the scientific research processes in which vaccines are produced were the most frequent theme. Claims such as a lack of sufficient scientific data on vaccines, the quick development procedure of vaccines, and the belief that the public is going to be experimental subjects for the vaccines were the main arguments in these themes.

*Example 1: Why do we get the vaccine that even [nation name] government does not use it for its own people? We are not experimental subjects.*

*Example 2: The only way to end the pandemic is to get vaccinated. However, no scientific evidence has been presented regarding the efficacy and the safety of the current vaccines. Without safety, no vaccine should be given to large populations.*

#### 3.2.2. Conspiracy Theories

There were claims stating that the pandemic is a set-up by some mysterious international powers, and that the vaccine is going to be used as a biological weapon to change the demographic structure.

*Example 1: Do you really believe all of these? They all tell you what globalists order them. First, it was supposed to end in 2021 summer, but now we are in 2021 winter, and they already extend it for the next ten years. I think it will end in 2071! My final call. What about those saying that vaccine is the light at the end of the tunnel? Vaccines are useless apparently.*

*Example 2: This is only the beginning. If we ain’t gonna stop all these today, their next steps might be even more dangerous. Don’t you get that the real matter is not COVID, vaccine, 5G, nor mask!*

#### 3.2.3. Suspicion towards Manufacturers

This theme refers to the expressions of suspicions towards the vaccine producer companies or countries.

*Example 1: While [nation name] gets the vaccines from [another nation name] for its citizens, they sell us their illegal vaccine that does not even complete phase 3.*

*Example 2: Today, I’ve heard rumors about, “[company name] and [nation name] vaccines are produced with active and passive viruses, respectively. And those who’ve gotten the [company name] are miserable right now.” I don’t know about their accuracy and I could not see any news about them. But, as I said, it’s all getting chaotic because they do not have any kind of safety mechanism.*

#### 3.2.4. Suspicion towards Health Authorities

Some tweets expressed suspicions against the credibility of health authorities. Dissatisfaction about countries’ methods of combating the pandemic and claims of secret relationships in the health sector were included in such tweets.

*Example 1: [scientist name] [company name] [vaccine brand] etc. You can show the dirty past of all vaccine producers and medical companies. You can countin all the doctors and their institutions as well. And all the governments that took side… Media, don’t be afraid. #GLOBALDECEPTION*

*Example 2: Dear minister, we don’t believe in you anymore. You’ve ruined the lives of all people and artisans. They are all #GLOBALDECEPTION We all are sick of your lies and do not trust in your vaccines. And, we are not gonna get any of them. [URL]*

#### 3.2.5. Undirected Distrust

There have been some statements that there are many uncertainties about vaccines, and that they cannot be trusted yet. There were claims stating that the vaccine is a lie, and it does not exist.

*Example 1: Lies about pandemic and institutions that fuel the fear among society… COVID might be a game or more of a trailer for a bigger game. Even the vaccination is a huge mystery.*

*Example 2: It should be all followed to see where this mutation stuff will be related to. I feel like it’s gonna be a reason for the mandatory vaccination instead of the second wave. Almost all of the vaccination companies have already stated that the vaccines are effective even when the virus has mutated.*

#### 3.2.6. Violation of Autonomy

Objections to the mandatory vaccination in terms of the privacy of the human body were included in this theme. Among the arguments presented were forced vaccination and the imposition of this decision by the national public authorities on behalf of citizens.

*Example 1: It is not obligated yet we are not happy with this vaccination imposition. #CitizensAreTheState*

*Example 2: So they all are gonna make debates through my body about the mandatory vaccination, but I am gonna shut up, not look it up and come into the line like a sheep. Is that so?*

#### 3.2.7. Unsafety

Tweets claiming that the vaccine has various harms on the body made up the “unsafety” theme. There were arguments that the vaccine has fatal effects, its side effects are severe, and it may even cause cancer.

*Example 1: Nobody knows the side effects of that vaccine on me. I prefer not having a vaccine that may harm or kill me, just for the sake of protecting others. And we all have a right to do this, do you get that? The vaccine or the experiment that’s gonna happen to me, I’ll make the call for that. What do you expect?*

*Example 2: If the vaccine will result in malformed births for the next generations. I am pretty sure it will be. (There are tons of examples for the malformed births in Africa and India because of the mRNA vaccines). In order to avoid its damages, the unvaccinated generation should not marry with the vaccinated generation. You can’t see the extent of its damages.*

#### 3.2.8. Non-Necessity

The “unnecessity of the vaccine” theme involves the claims that vaccines are not necessity in combating pandemics. The statements within this theme included arguments that the disease is mild, the mortality rate is low, and that strong natural immunity is sufficient.

*Example 1: Did you even end the flu with vaccine? You cannot vaccinate a virus that’s mutated. All we need is to have a strong immune system. That’s it.*

*Example 2: We do not wanna get vaccination or something like that. Does anybody hear our voice??? I’ve survived from corona at home without any medicine. My relative that had a hard time during their recoveries also survived from it at thome. The half of my country people have survived it. I don’t wanna get a vaccination for something that I’ve already gotten the immunity for.*

#### 3.2.9. Ineffectiveness

The claims that vaccines are ineffective against the virus, and therefore against the pandemic has been frequently expressed. Among these statements were the possibility of getting sick despite the vaccine, that it did not work against mutant viruses, and that it did not prevent transmission or death.

*Example 1: I think the vaccination is not the way to end the disease. Whats’ important is that we need to develop medicines that can lead people to survive from this disease easily. The flu vaccinations are not%100 protective and they’re never gonna be. Everybody gets caught a flu and gets well with a medicine. That’s the only solution that we should focus.*

*Example 2: Does the COVID-19 vaccine not working well? A doctor who got the vaccine 6 days ago just caught the corona virus again.*

#### 3.2.10. Influential People

Statements were made on opinion leaders or celebrities who got vaccinated or declared that they would not. There were expressions such as the low number of people who stated that they would be vaccinated, the majority of those who stated that they would not, and the distrust of someone who endorsed the vaccine.

*Example 1: [Controversial celebrity name] praised the [nation name] vaccine and said that those who discredit it create negative perception. I hope you understand why we should question this vaccine.*

*Example 2: Don’t fool yourself, none of them have gotten the vaccine because there is no such a thing as COVID. You don’t wanna get this. Do you really think that they all get the vaccine?*

#### 3.2.11. Pandemic Denial

This theme included another frequent claim regarding the denial of the existence of the pandemic. There were claims that the disease or virus did not actually exist, it was a lie, and fake.

*Example 1: Although it might be a regular vaccine, there is no need for it because there is no such a disease. But, how are you really gonna believe whether those people in the media saying that they got vaccinated? Also, its effects should last 4–5 years at least.*

*Example 2: Look at these photos that were just taken. Also in Wuhan… Look, how China is messing with you al. Even there is a virus for those tribe countries that have almost no people living in them, China with 2 billion people is joking with the world. No vaccine, no treatment, yet we are done with virus, they says. WAKE UP PEOPLE THERE IS NO VIRUS*

#### 3.2.12. Financial Interests of Manufacturers

Under this theme, there were some claims that the vaccines emerged completely because of financial concerns. The reasons presented to suppose these statements included claims that the vaccine was commercial, expensive, and for-a-fee, and that the goal was to earn money.

*Example 1: Those people used to say that vaccines will be free but now trying to make profit out of them. They’ll even get taxes.*

*Example 2: They all are really trying to make profit out of it. At the beginning, they all said that vaccines will be free, and now there are rumors saying that one dose will be 10 dollars.*

#### 3.2.13. China’s Oppression of Uighurs

A few tweets were about the relationship between China’s East Turkestan policies and the vaccination process. There were claims that vaccines were provided to Turkey by a country that also persecuted the Uighurs and a commercial relationship with this country would mean betrayal of them.

*Example 1: Things have done to our Uighur Turkish brothers are never ending. Also, we are contributing to the Chinese economy by taking the vaccination from them although the Chinese government is the responsible for all pressures and tortures. These vaccinations are betrayal to our Uighurian brothers #UighursCannotBeRepatriated*

*Example 2: Let’s stop all the fuss and I am not gonna get a vaccine or not even let them into my apartment who gathers Uighurians into more than 500 different camps under the so-called a training program*

#### 3.2.14. Religious Beliefs

There were also those who objected to vaccinations on the basis of religious beliefs. These people claimed that the vaccines were not halal because of their ingredients.

*Example 1: We are not against the vaccine. We are against the vaccines that contain haram stuff. We wanna get halal drug and halal vaccine. #Vaccine #Drug #Halal #Local #National*

*Example 2: How’s it gonna be permissable both for them and for muslims? Whats the difference if we all gonna get that piggy foetus mRNA vaccine that gonna change our genetic codes? But, see the Pope does not even wear a mask. But of course it should be an exception because mask is a symbol of slavery and not gonna work out for them.*

## 4. Discussion

The herd immunity level required to alleviate the COVID-19 pandemic can only be achieved with widespread vaccination. However, anti-vaccination movements have increased in recent years and have surged even more during the pandemic [21].

This study is the first Twitter-based qualitative content analysis in Turkish on COVID-19 anti-vaccine and hesitancy. We analyzed tweets on Twitter during the delivery of the first batch of vaccines to Turkey. Among all vaccine-relevant tweets, 22.6% of them mentioned the name of a COVID-19 vaccine and 22.0% included at least one anti-vaccination theme.

CoronaVac was the most frequently mentioned vaccine and was mostly referred to as “the Chinese vaccine”. Interestingly, people described this vaccine mostly by its country of origin. Comirnaty was the second most frequently mentioned vaccine and was mostly referred to as the Pfizer-Biontech vaccine, which is the company of origin.

During the analysis of the anti-vaccination contents of the tweets, we identified fourteen major themes. “Poor scientific process” was the most predominant theme, and it was followed by the themes of “conspiracy theories” and “suspicion towards manufacturers”. The themes of “poor scientific process” and “suspicion towards manufacturers” and “poor scientific process” and “suspicion towards health authorities” tended to occur together. In addition, “suspicion towards health authorities” and “conspiracy theories” themes tended to occur together (Figure 3). We believe that general mistrust underlies these combinations of themes.

We are of the opinion that the “poor scientific process” theme’s frequency was higher than others because of the fast-tracked vaccine development process due to the urgent need of the vaccine to reduce the effects of the pandemic. A similar concern that “The vaccine was developed too quickly” emerged in Berry et al.’s study in United States [21]. Furthermore, the lack of information during the time period of interest in our study about the results of phase 3 trials of CoronaVac, which was the only COVID-19 vaccine that was delivered to Turkey at the time, might have increased the prevalence of arguments in line with this theme in our sample.

The second most frequent theme was “conspiracy theories”. The arguments in relation to this theme are similar to those observed in Sallam et. al’s online questionnaire [22] conducted in Arab Countries and Ortiz-Sánchez et al.’s systematic review [9], which was performed on certain databases to analyze networks’ information about the anti-vaccine movement. Sallam et al.’s questionnaire results showed that 59.5% of the respondents believed that COVID-19 is a man-made virus and 40% of them thought that it was made to force everyone to get the vaccine [22]. According to Salali and Uysal’s research that was conducted in the United Kingdom (UK) and Turkey using an online survey, 18% in Turkey and 12% in the UK thought that the origin of the virus was artificial [23]. In our study, we found that 3.6% of the tweets contained a comparable claim. Moreover, Nuzhath et al.’s content analysis, which was conducted on Twitter, showed similarities with our findings. That study also identified the theme of “conspiracy theories” and it was found to be the second most frequent theme, as in our study. Our findings parallel their findings in that this theme involved some theories such as that the “Vaccine is being developed to limit or control population size”, “Vaccine will contain microchip or tracking device”, “5G/3G technology related to COVID-19 infection and vaccine”, and “Vaccine makers created COVID-19” [24]. A similar concern was also found in Berry et al.’s study, regarding the “microchip” suspicion [21].

The “suspicion towards manufacturers” theme included the expressions of suspicions towards the companies or countries that produced the vaccine. The theme of “suspicion towards health authorities” included dissatisfaction about countries’ methods of combating the pandemic and claims of secret relationships in the health sector. Similar themes were found under the theme of “mistrust in health institutions” in a rapid literature review that included articles on vaccine confidence, trust, and hesitancy published between 2004 and 2014 in Europe [25], and the theme of “authority figures” was identified in a content analysis of tweets conducted in Canada [26].

In a study conducted in Australia to examine the public acceptance of a safe and effective vaccine developed for COVID-19, it was found that confidence in the health system and the government was positively associated with vaccine acceptance. In this regard, an increase in confidence in health authorities would have a positive impact on the acceptance of the COVID-19 vaccines, as well as other types of vaccines [27].

Another frequent theme in our analysis was “violation of autonomy”, which involves objections to obligatory vaccination based on the view that human bodies are private property. When a population is at risk, such as during a pandemic, collective interests are prioritized over individual ones. Implementations such as quarantine, isolation, and social distancing aim at prioritizing collective interests yet limit the individual’s freedom and autonomy [28]. This threat to individual autonomy may be one of the reasons that provokes people to share their concern about mandatory vaccination.

The seventh most frequent theme in our analysis was “unsafety”, which parallels the “safety” theme, which was found to be the most frequent theme in the content analysis of tweets in Canada in an exploration of public attitudes toward vaccination [26]. The themes of “non-necessity” and “ineffectiveness” showed similarities with the determinants “not required” and “vaccines not effective” identified in the rapid literature review that was conducted in Europe before the COVID-19 pandemic [25]. The “non-necessity” theme is also similar to the argument that developing immunity against coronavirus is not necessary as COVID-19 infections result in low mortality rates, which emerged in the Twitter-based research of Nuzhath et al., conducted in English [24].

Another frequent theme was “pandemic denial”, which may negatively affect not only vaccination attitudes, but also the compliance to precautions such as maintaining social distance and wearing masks.

Previous studies on vaccine hesitancy and anti-vaccination attitudes have not identified the origin country of a vaccine as a common concern. We found in a few tweets the theme of “China’s oppression to Uighurs” was more specific than other studies carried out on this subject. We suggest that this theme emerged from the vaccine CoronaVac being usually referred to as “the Chinese vaccine” and reflected a nationalist concern against China’s East Turkestan policies, where Uighur people lived.

The least frequent theme in our analysis was religious beliefs. Islam is the largest religion in Turkey and is not in contradiction with COVID-19 vaccines. In addition, during the vaccination campaign in Turkey, the Turkish Directorate of Religious Affairs declared that Muslims needed to comply with the precautions, which included vaccination, during the pandemic [29]. Moreover, no relationship was found between the religious beliefs and neither negative nor positive attitudes towards COVID-19 vaccines were identified in Kilic et al.’s research with Turkish participants [30].

**Figure 4:**
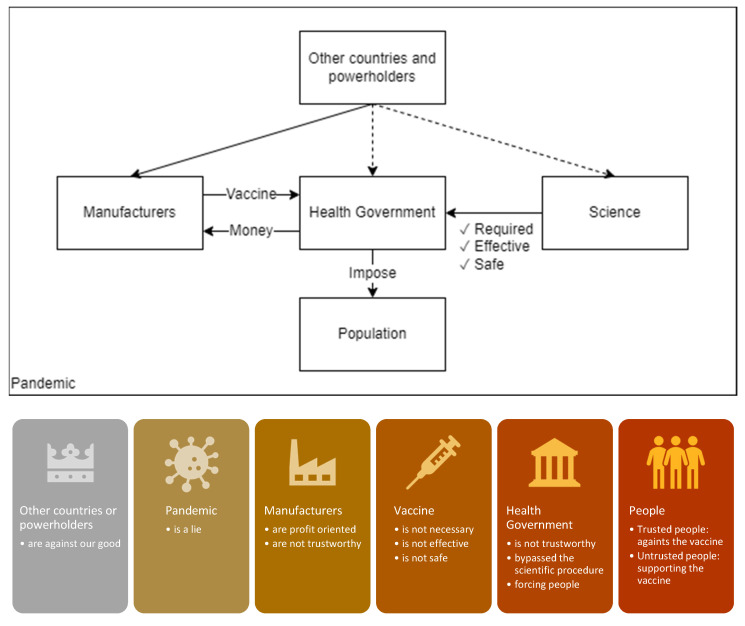
Synthesis of vaccine hesitancy attitudes in tweets.

The emerging themes that describe the vaccine-hesitant attitudes in our sample of tweets are synthesized in Figure 4. Vaccine-hesitant people often expressed a concern regarding an outer threat, which sometimes targeted vaccine manufacturing countries but remained ambiguous in other instances. Some of the vaccine-hesitant informants in our sample did not believe that there was a pandemic at all and viewed the pandemic to be staged or planned by powerholders. Most of our informants expressed a distrust in vaccine manufacturers. This distrust was partially due to the manufacturers’ financial interest in manufacturing vaccines but could also be related to conspiracy theories. As a combined result, hesitancy has arisen regarding the necessity, effectiveness, safety, procurement, and enforcement of vaccines and vaccination. Being or feeling distant from the decision-making processes regarding vaccination programs also contributes to this hesitancy. Although some tweets imply a distrust of modern science as an institution, the bypassing of lengthy scientific procedures in vaccine development processes was a more influential reason for hesitation. These scientific concerns might be interpreted as an improvement area and the trust of those questioning public health governance and policies regarding their compliance with science can be established through more effective science communication. The perception of an untrustworthy context such as during the initial stages of vaccination against COVID-19 leads to people being more easily influenced by the behavior of other people around them, which suggests that mending public trust is an important part of intervention against vaccine hesitancy.

Even though these results seem to include diversely motivated arguments in support of the anti-vaccination and vaccine hesitancy movements, it is important to note that the tweets in our sample were written during the delivery of the first batch of vaccines to Turkey. During that period, vaccines were on the front burner, with many uncertainties. Some profiles might share emotional tweets on the hot topic of vaccines in order to attract other people’s attention and gain social interactions, and this might present a skewed and pessimistic image that does not reflect the public experience. Our opinion is supported by Germani and Biller-Andorno’s finding that anti-vaccination supporters produced more content than pro-vaccination profiles; however, anti-vaccination supporters on Twitter had fewer original arguments with less received average engagement than the tweets produced by pro-vaccination profiles [31]. Furthermore, in accordance with this discrepancy, the percentage of fully vaccinated citizens over 18 years of age reached 78.70% during the vaccination campaign in Turkey and this number is rising [32] despite the prevalence of the vaccine-hesitant content in our study.

Social media is the most preferred platform for obtaining information on almost every topic in our daily life. Today, the problem is not in accessing information, but in distinguishing whether that information is correct or not. In the post-truth era, even obvious facts are denied through various deceptions and manipulations. The vast majority of anti-vaccine themes in our study were issues relating to manipulating objective information. Lack of information or infollution provide the basis for the observed themes, in which the most prominent keywords were distrust, suspicion, conspiracy, and denial. Moreover, the most frequently stated reasons for anti-vaccination in the literature are similar to those in our study [9,33]. However, almost all of these doubts can be removed with scientific proofs. Although new studies published every day have repeatedly proven the positive effects of vaccines, it is thought-provoking that the underlying reasons for opposition still prevail. The common findings revealed in the studies conducted among different populations show that both the official authorities and the industry should conduct their own self-criticism. Instead of ignoring doubts and concerns, they should address them and find solutions to eliminate them. From this perspective, it is critical for authorities to allow the people to participate in the decision-making processed, to provide transparency, give clear information, and undertake appropriate risk communication for the population.

A number of potential limitations need to be considered in our research. We first conducted the content analysis on original Turkish texts, then translated them into English. The meaning of the original tweets and the misspellings may be lost in translation. Furthermore, this study included tweets from a specific one-month period, during the delivery of the first batch of vaccines to Turkey, and it did not include the time during vaccination. It is also important to analyze the changes in attitudes during the pandemic period. In a study by Hyaland et al., it was found that there were fluctuating changes over time [34]. In this sense, the repetition of this research in different time periods is important in terms of determining the different contents of hesitation in specific time periods.

Our research is also limited by our keywords. Since we collected the tweets based on a limited set of keywords, we might not have caught more complex or misspelled occurrences of the word “vaccine”. Finally, our research was limited to Twitter and might have included tweets from other Turkish-speaking communities because analysis of the geographic origin of tweets was not feasible due to there only being a small number of tweets including a geocode. However, social media data tended to aggregate with real-life events, and we collected data in the midst of the vaccine roll-out in Turkey. Thus, it was expected that the vast majority of tweets were from Turkey. Though we conducted this content analysis among Turkish tweets, themes showed similarities with other studies conducted in different countries and languages. Thus, our findings may be helpful for other countries’ health authorities in regard to COVID-19 vaccine hesitancy.

There are also different personal and environmental factors that are possibly associated with vaccine acceptance. In this study, the arguments against the vaccine were discussed, and the individual characteristics of the people who were hesitant were not evaluated. Determining these factors could provide a guide for focused interventions.

Cristea et al. have showed a correlation between positive attitudes towards the COVID-19 vaccine and people’s perceptions of vaccine acceptance among acquaintances or the general population [35]. Thus, we can suspect the presence of a relationship between people’s negative attitudes against the vaccine and exposure to a range of negative attitudes from social media. Our findings can inform further studies on this relationship.

In addition to this research, the questions of how information and opinions about vaccines are spread on social media and how they influence people should be examined in further research. A study conducted in Dutch society in 2019 identified narratives in vaccine debates on Twitter and interactions between different types of communities [36]. This study found that groups with negative contents had more interaction with other communities, which is an important finding in terms of understanding the spread of anti-vaccine arguments. Determining how these interactions occur during the pandemic period is also important for planning the interventions. Silva et al. demonstrated how to use people’s opinions and networks from social media to inform a mathematical model of COVID-19 transmission and support policy making [37]. Qualitative studies such as ours can be used to improve the inputs for those kinds of models.

## 5. Conclusions

In conclusion, anti-vaccination and vaccine hesitancy supporters can easily share their arguments on their social media profiles and build echo chambers. Thus, monitoring these kinds of movements on social media is integral to planning a better health communication strategy. It is well known that vaccine hesitancy and anti-vaccination attitudes may negatively influence the population’s health, especially during a pandemic, and social media content is an important source of early information about such attitudes. The analysis of social media content may be helpful for public health professionals in identifying the major issues and organizing timely preventive measures. Social media is becoming more and more influential in health-related issues, and in many others. Authorities should adapt their traditional communication methods, become more visible on these platforms, and should take a proactive approach, especially during such milestone events.

Although it is known that vaccine-hesitant people may have trust problems and faulty reasoning, it is more important to understand how they feel. The findings of this study imply that they feel like they are under threat. They have been excluded from all vaccine-related decision-making processed, such as those concerning whether vaccines are required, effective, or safe; decisions about where to import vaccines from, and whether vaccination should be imposed upon the entire population. They express feelings of not being heard and being a passive object in relation to what is happening. Including them in decision-making processes either personally or through trusted representatives would be helpful in order to build public trust in vaccines and vaccination.

## Figures and Tables

**Table 1:** Characteristics of the user accounts.

	*n*	%
Verification status		
Not verified	973	97.3
Verified	27	2.7
User type		
Personal or others	887	88.7
Organizational	113	11.3
Total	1000	100.0
	**Median (IQR)**	
Duration of Twitter use (year)	4.0 (1.0–8.0)	
Number of followers	276.5 (55.0–603.8)	
Number of tweets published	3163.5 (1561.5–13,951.0)	

**Table 2:** Characteristics of the tweets.

	*n*	%
Presence of a visual		
Yes	116	11.1
No	925	88.9
Presence of an URL		
Yes	219	21.0
No	822	79.0
Presence of a hashtag		
Yes	123	11.8
No	918	88.2
Relevancy with the vaccine		
Irrelevant	99	9.5
Relevant	942	90.5
Total	1041	100.0
	**Median (IQR)**	
Number of tweets per day		
Before arrival of vaccines (11 Dec–29 Dec)	29.0 (25.0–40.0)	
After arrival of vaccines (30 Dec–10 Jan)	27.0 (24.0–29.0)	
Total	29.0 (24.5–38.5)	

**Table 3:** Tweet contents, including vaccine names and anti-vaccination themes.

Contents	*n*	%
Vaccine names		
Name of a COVID-19 vaccine	213	22.6
Name of other vaccines *	25	2.7
No vaccine name	705	74.8
Total	942	100.0
Anti-vaccination themes		
Present in tweets	207	22.0
Not present in tweets	735	78.0
Total	942	100.0

* Influenza, pneumonia, rabies, multiple sclerosis, tetanus, smallpox, polio, cholera, human papillomavirus. One tweet mentioned both COVID-19 and multiple sclerosis vaccines.

**Table 4:** Distribution of the vaccine names in tweets that mentioned a COVID-19 vaccine.

Names of the Vaccines	*n* *	%
CoronaVac mentions	109	51.2
Chinese vaccine	90	42.3
Sinovac	19	8.9
CoronaVac	4	1.9
Comirnaty mentions	57	26.7
Pfizer-Biontech	54	25.4
German vaccine	5	2.3
Comirnaty	1	0.5
Moderna mentions	14	6.6
Moderna	12	5.6
American vaccine	4	1.9
National (Turkish) vaccine mentions	12	5.6
mRNA vaccine mentions	8	3.8
AstraZeneca mentions	7	3.3
Oxford	4	1.9
AstraZeneca	3	1.4
Sputnik V mentions	5	2.3
Russian vaccine	4	1.9
Sputnik V	1	0.5
Other COVID-19 vaccine mentions	14	6.6
Total	213	100.0

* Sum of the numbers is not equal to the total and subtotals because some tweets included more than one vaccine name.

**Table 5:** Distribution of anti-vaccination themes.

Themes	*n*	%
Poor scientific process	45	21.7
Conspiracy theories	34	16.4
Suspicion towards manufacturers	32	15.5
Suspicion towards health authorities	27	13.0
Undirected distrust	26	12.6
Violation of autonomy	25	12.1
Unsafety	23	11.1
Non necessity	21	10.1
Ineffectiveness	17	8.2
Influential people	14	6.8
Pandemic denial	11	5.3
Financial interests of manufacturers	9	4.3
China’s oppression of Uighurs	8	3.9
Religious beliefs	3	1.4
Total *	207	100.0

* Sum of the numbers is not equal to the total because 71 tweets include more than one theme (See also Figure 3).

## Data Availability

Data is available upon request from corresponding author.

## Supplementary Materials

The following are available online at https://www.mdpi.com/article/10.3390/vaccines10020161/s1, File S1: Complete search query, File S2: Original Turkish texts of quotations.
