# Supplementary material for: Vaccine Hesitancy and Anti-Vaccination Attitudes during the Start of COVID-19 Vaccination Program: A Content Analysis on Twitter Data
Source: Vaccines (Basel). 2022 Jan 21;10(2):161. doi: 10.3390/vaccines10020161 (PMC8876163; doi:10.3390/vaccines10020161)
Supplement: Supplementary file 1 [file vaccines-10-00161-s001.zip › S1_File.pdf]

S1: The search query

The original search query used for data collection:

“aşı OR aşıyı OR aşıya OR aşıda OR aşıdan OR aşının OR
aşılar OR aşıları OR aşılara OR aşılarda OR aşılardan OR aşıların OR
aşısı OR aşısını OR aşısına OR aşısında OR aşısından OR aşısının OR
aşıları OR aşılarını OR aşılarına OR aşılarında OR aşılarından OR aşılarının”

English translations for search terms

aşı: the vaccine
aşıyı: the vaccine
aşıya: to the vaccine
aşıda: in the vaccine
aşıdan: from the vaccine
aşının: of the vaccine

aşılar: the vaccines
aşıları: the vaccines
aşılara: to the vaccines
aşılarda: in the vaccines
aşılardan: from the vaccines
aşıların: of the vaccines

aşısı: [noun] vaccine
aşısını: the [noun] vaccine
aşısına: to the [noun] vaccine
aşısında: in the [noun] vaccine
aşısından: from the [noun] vaccine
aşısının: of the [noun] vaccine

aşıları: [noun] vaccines
aşılarını: the [noun] vaccines
aşılarına: to the [noun] vaccines
aşılarında: in the [noun] vaccines
aşılarından: from the [noun] vaccines
aşılarının: of the [noun] vaccines
