# Supplementary material for: Vaccine Hesitancy and Anti-Vaccination Attitudes during the Start of COVID-19 Vaccination Program: A Content Analysis on Twitter Data
Source: Vaccines (Basel). 2022 Jan 21;10(2):161. doi: 10.3390/vaccines10020161 (PMC8876163; doi:10.3390/vaccines10020161)
Supplement: Supplementary file 1 [file vaccines-10-00161-s001.zip › S2_File.pdf]

S2: Original texts of quotations

Original Turkish texts of quotations for each theme are provided below.

Poor scientific process

Örnek-1: [Ülke adı] kendi halkına yapmadığı aşıyı bizler neden yapıyoruz. Biz turk milleti kobay değiliz

Örnek-2: Pandeminin sona ermesi için tek yol olarak aşılar gösteriliyor. Oysa mevcut aşıların koruyucu olduğuna ve insan sağlığı açısından güvenilir olduğuna dair hiç bir bilimsel veri sunulmamış durumda. Güvenilir olduğu ispatlanmadan, bu aşılar kitlelere uygulanmamalıdır.

Conspiracy theories

Örnek-1: Siz hala bunlara inanıyor musunuz? Küreselciler ne emir verirse onu söylüyorlar, 2021 yaz aylarında bitecek,sonra 2021 kışı oldu, şimdi 10 yıla uzatmışlar⍰⍰ bence 2071de biter! Son kararım. Eee hani tünelin sonundaki ışık aşıydı? Aşılar da işe yaramıyor belli⍰

Örnek-2: Daha bu işin başlangıcı, Bunları bugün burada durduramaz isek, Allah fırsat vermesin, bir sonraki adımları daha tehlikeli olabilir.

Mesele, CoVID, aşı, 5G, maske değil anlamadınız mı!

Suspicion towards manufacturers

Örnek-1: [ülke adı] kendi vatandaşı için aşıyı [diğer ülke adı]'dan alsın, bize de faz 3 aşamasındaki henüz yasal olmayan aşıyı satsın

Örnek-2: Bugün de "[şirket adı] asisi aktif virüsle, [ülke adı] asisi pasif virüsle yapilmis; [şirket adı] olanlar cok kotu etkilenmiş" rivayetlerini duydum, nedir dogruluk payi hicbir fikrim yok. Biraz arastirdim pek bir haber goremedim ama dedim ya, guven mekanizmasi olmadigi icin her sey kaos gibi su an.

Suspicion towards health authorities

Örnek-1: [bilim insanı adı] ([şirket adı] [aşı markası] vs vs) ve diğerleri… Bütün aşı ve ilaç firmalarının kirli geçmişlerini gösterebilirsiniz. Bütün doktorların ve hizmet ettikleri kurumları da ifşa edebilirsiniz. Bütün devletlerin hangi tarafta olduğunu da... Korkak olma medya.

Örnek-2: sayın bakan artık size inanmıyoruz milletin, esnafın hayatını alt üst ettiniz. #KURESELALDATMACA bunlar. sizin yalanlarınızdan bıktık artık. aşılarınızada güvenmiyoruz aşıda vurulmayacagiz [URL]

Undirected distrust

Örnek-1: Pandemi yalanları ve korku pompalayan kurumlar… Covid bir oyun belkide daha büyük bir oyunun önizlemesi… Aşı bile koca bir muamma…

Örnek-2: Takip edilmeli, göreceğiz bu mutasyon işini neye bağlayacaklar. 2. Dalgadan ziyade, sanki aşıyı zorunlu kılmak için bir gerekçe olacağı gibi; zira aşı firmalarının hemen hepsi, aşıların mutasyona uğramış virüs üzerinde de etkili olduğunu ilan ettiler..

Violation of autonomy

Örnek-1: Zorunlu değil ama mecburi aşı dayatmasına razı değiliz #Vatandasdevlettir

Örnek-2: Benim bedenim üzerinden zorla aşı konusu tartışmaya açılacak, ama ben susacağım, araştırmayacağım ve koyun gibi sıraya gireceğim öyle mi ?

Unsafety

Örnek-1 : O aşının bana yapacağı yan etkiyi kimse bilmiyor başkasını koruyacağım diye bana zarar verebilecek yada beni öldürecek aşıyı seçmek yerine seçmemeyi seçerim bunada hakkimiz var anladın mı bedenime yapılacak aşıya deneye ben karar veririm biz karar veririz ne demek oyle

Örnek-2 : Aşı sonucu, ileri nesillerde sakat doğumlar olursa.. Ki olacak.. (mRNA aşılarının afrika ve hindistanda binlerce sakat doğum örneği var) Zararından tamamen uzaklaşmak için, aşı olmamış nesil, aşı olmuş nesille evlenmemesi lazım. Gör ki, zararı nerelere kadar gidiyor..

Non necessary

Örnek-1: Gribi aşıyla bitirdin mi ki? Mutasyon virüse aşı olmaz Güçlü bağışıklık gerekli O kadar

Örnek-2: İstemiyoruz aşı olmak falan olmak istemiyoruzzz. SESİMİZİ DUYAN VARMIIII???? Ben korona oldum evde ilaçsız atlattım. Akrabalarımdan ağır geçirenler evde atlattı. Ülkemin yarısı atlattı. Bağışıklık kazandığım birşeyin AŞISINI OLMAK İSTEMİYORUM.

Ineffectiveness

Örnek-1: Aşı hastalığı bitirecek bir yöntem değil bence. Önemli olan hastalığı kolay atlatmamızo sağlayacak ilaçların geliştirilmesi. Grip aşıları %100 koruyucu değil bu da olmayacak. Herkes grip olur ilaçla iyileşir. Bu kıvama gelmesi tek çözüm.

Örnek-2: Kovid-19 aşısı işe yaramıyor mu? 6 gün önce aşı olan doktor korona virüse yakalandı

Supporting and opposing people

Örnek-1: [Tartışmalı bir ünlü adı] [ülke adı] aşısını övmüş ve kötüleyenler algı yaratıyorlar demiş umarım neden bu aşıyı sorgulamamız gerektiğini anlamışsınızdır.

Örnek-2: Hicbiri aşı olmadi kandırmayın kendinizi çünkü covit diye birşey yok bunu anlamak istemiyorsunuz sizce bu aşıyı olurlar mı

Pandemic denial

Örnek-1: Normal aşı da olsa gerek yok olmayan bi hastalık için,ama nası inancaksın aşı vuruldum diye madyaya çıkanların gerçekten aşı olduğuna,ayrıca 4 5 sene takip edilmeli etkileri.

Örnek-2: Bakın bu fotoğraflar heni çekildi.. Hemde wuhan'da.. bakın Çin sizinle nasıl dalga geçiyor.. insanın olmadığı kabile ülkelerinde bile virus varken.. 2 milyarlık çin dünyayla dalga geçiyor aşı yok tedavi yok virusu bitirdim diyor.. UYANIN ARTIN VİRUS YOK

Financial concerns

Örnek-1: Asi herkese ucretsiz uygulanacak diyenler simdide ondan kar etme derdinde kdv alacak

Örnek-2: işi iyice ticarete bağladılar ahmet hocam ilk başta ücretsiz dediler sonra 1 doz aşı 10 dolar olacak diye söylentiler var milleti galyaneye getirmeye çalışıyorlar halbuki öyle bir hastalık yok uydurmaca neymiş efendim nüfüs planlaması milleti kandırıyorlar...

Uighurs

Örnek-1: Uygur'lu Türk kardeşlerimize yapılanlar bitmiyor. Üstelik biz bu baskıları, işkenceleri yapan Çin devletinden aşı alarak ekonomilerine destek sağlıyoruz. Aldığımız bu aşılar Uygur'lu kardeşlerimize yapılan bir ihanettir. #UygurlarİadeEdilemez

Örnek-2: Onu bunu bırakında, Uygur'lu Türkleri sözde eğitim adı altına, 500 taneden fazla kampta toplayan bir ülkeden bırak aşı almayı , kapımdan içeri sokmam.

Religious beliefs

Örnek-1: Biz aşıya karşı değiliz. Haram içerikli aşılara karşıyız. Biz helal ilaç ve helal aşı istiyoruz. #Aşı #İlaç #Helal #Yerli #Milli

Örnek-2: onlarada caiz biz müslümanlarada caiz domuz lu fetüslü MRNA lı genetik kodlamızı değiştirecek aşılar nerde kaldı farkımız.papa maske takmıyor o yönden farkı olsun tabiki kölelik alâmeti maske onlara yakışmaz.
